# Effects of polymers on the properties of hydrogels constructed using sodium deoxycholate and amino acid†

**DOI:** 10.1039/c8ra00171e

**Published:** 2018-02-27

**Authors:** Yi Guo, Ruijin Wang, Yazhuo Shang, Honglai Liu

**Affiliations:** Key Laboratory for Advanced Materials, School of Chemistry & Molecular Engineering, East China University of Science and Technology Shanghai 200237 China shangyazhuo@ecust.edu.cn hlliu@ecust.edu.cn +86-21-64252767 +86-21-64252921

## Abstract

The gelation behavior and properties of sodium deoxycholate (NaDC) and l-aspartic acid (Asp) in aqueous solution were investigated in detail at 25 °C. The linear polymer poly(2-(2-methoxyethoxy)ethyl methacrylate-*co*-oligo-(ethylene glycol) methacrylate) (P(MEO_2_MA_90_-*co*-OEGMA_10_)) and star-shaped polymer poly(2-(dimethylamino)ethyl methacrylate-*b*-2-(2-methoxyethoxy)ethyl methacrylate) (CDPDPM) were introduced in NaDC/Asp hydrogels for exploring the effects of polymers on the properties of NaDC/Asp hydrogels and the mechanism underlying gelation processes by polymers was proposed. The hydrogels were characterized by phase behavior observation, polarized optical microscopy (POM), cryogenic scanning electron microscopy (cryo-SEM), X-ray powder diffraction (XRD), Fourier transform infrared (FT-IR) spectroscopy and rheological measurements. Moreover, the adsorption performances of hydrogels with and without polymers to methylene blue (MB) were studied using a UV-vis spectrometer. The results indicated that the transition from sol to gel state was observed with an increase in the Asp concentration in the system. Both linear and star-shaped polymers can participate in the formation of a gel network structure, so that the density of network structure and the mechanical strength of hydrogels increased. Furthermore, it was found that the viscoelasticity of the CDPDPM-containing hydrogel was much higher than that of the P(MEO_2_MA_90_-*co*-OEGMA_10_)-containing hydrogel under the same condition, indicating that CDPDPM performed better in strengthening the network structure of the hydrogels than P(MEO_2_MA_90_-*co*-OEGMA_10_) due to the special structure that provided more binding sites for hydrogen bonding and stronger hydrophobicity that inhibited the swelling and dissolution of hydrogels. On coming in contact with the MB solution, the CDPDPM-containing hydrogel can adsorb MB and maintain the hydrogel state for recycling. On the contrary, the NaDC/Asp hydrogel dissolved and P(MEO_2_MA_90_-*co*-OEGMA_10_)-containing hydrogel collapsed in the MB solution. The properties of the hydrogels are expected to be tailored by introducing polymers with different properties, including the charge numbers, the number of available binding sites, and hydrophobic properties.

## Abstract 2

Polymer can participate in the formation of hydrogel network structure and provide a lot of binding sites, leading to an enhancement of the mechanical strength of the hydrogels.

## Graphical abstract

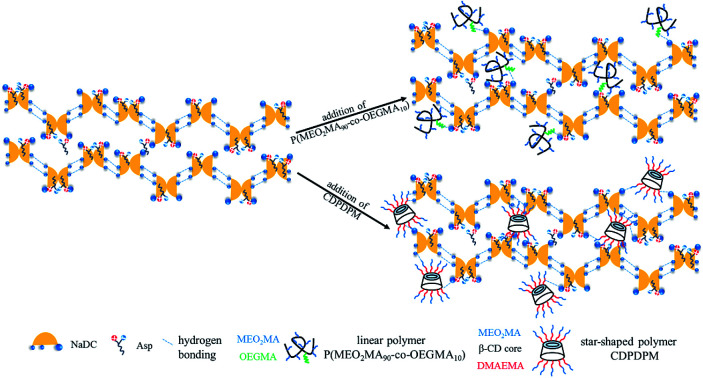

## 1. Introduction

Supramolecular hydrogels, as an important class of soft matter, have received considerable attention in recent years because of their remarkable potentials in fields of biomaterials,^1^ drug delivery,^2,3^ sensing,^4,5^ tissue engineering,^6^ separation science,^7^*etc.* Generally, the supramolecular hydrogels are derived from the self-assembly of low-molecular-weight gelator *via* various noncovalent interactions, such as hydrophobic interactions, hydrogen bonding, van der Waals forces, and electrostatic interactions.^8–10^ The gelators are usually based on small organic molecules.^11^ Among these gelators, the biocompatible and biodegradable species are particularly popular for constructing hydrogels, such as nucleobases,^12^ bile salts,^13^ carbohydrates^14^ and peptides,^15^ as they can be safely used in biological applications.^16^

Bile acids and their salts belong to not only important biological surfactants, but also small-molecule biological gelators that can act as solubilizers of cholesterol, bilirubin, and various fat-soluble vitamins in the intestine of vertebrates, including humans.^17,18^ Unlike the typical surfactant that carries a polar head and a nonpolar tail, the bile salt is well known as a facial amphiphile and possesses a rigid steroid backbone comprising hydroxyl groups and a carboxyl groups on the polar face and methyl groups on the nonpolar face. Due to the special structure, bile salts can self-assemble in aqueous solutions to form special nanostructures including nanofibers and nanohelixes that have the potential to further cross-link to form gels.^19,20^ Sodium deoxycholate (NaDC), as one of the most studied bile salt hydrogelators, is sensitive to pH and yields hydrogels at slightly alkaline pH.^21^ However, the viscoelastic and mechanical properties of NaDC hydrogels are not strong enough. Thus, some additives are needed to construct composite hydrogels and improve their function. Numerous studies have been performed on the effects of salts, pH, metal ions, and amino acids on the properties of NaDC hydrogels.^20,22,23^ The existing results explicitly elucidate how these additives essentially affect the properties of NaDC hydrogels. Jover *et al.* prepared NaDC hydrogels in a phosphate buffer solution (PBS) and reported that the viscoelasticity of the hydrogel from NaDC in PBS increased drastically with an increase in NaCl concentration. They found that decreasing the pH or increasing the ionic strength of aqueous solutions of NaDC led to the protonation of the carboxylate group, and consequently strengthened the hydrogen bonding and weakened the electrostatic repulsion between the polar faces of the NaDC molecules. The intermolecular hydrogen bonds between carboxylic acid groups may result in gelation.^24^ Zhang *et al.* prepared supramolecular hydrogels in mixtures of NaDC and amino acids (glycine (Gly), alanine (Ala), lysine (Lys) and arginine (Arg)) in different pH buffer solutions. The results have shown that the introduction of Ala and Gly strengthens the NaDC hydrogels due to its suitable size and hydrophilicity, which endows them the probability of creating more linkages between the NaDC molecules, while the addition of Lys and Arg could break the hydrogen bonds and weaken the formation of gels.^25^ Wang *et al.* designed a type of luminescent hydrogels through supramolecular self-assembly of biological surfactants (NaDC) and lanthanide salt (europium nitrate (Eu(NO_3_)_3_)) and reported that the mechanical strength of the hydrogels increased with an increase in the concentration of NaDC and Eu(NO_3_)_3_.^26^ Clearly, hydrogen bonds are the foundation of forming hydrogels and the properties of hydrogels are primarily determined by the amount of hydrogen bonds formed in studied systems.

Polymer is a chemical compound composed essentially of a large number of repeating structural units and has been widely used in numerous fields such as medicine, food, and electronics.^27–29^ The adjustability of structure, chemical composition as well as polymerization degree of polymers endows it potential to be used in the gel forming system. On the one hand, the number of hydrogen bonds formed in the system can be altered by introducing special functional groups or changing the number of certain repeating units in the polymer. On the other hand, the mode of hydrogen bond arrangement can be tailed by adjusting the molecular configuration of polymers. Polymers should have significant effects on the properties of hydrogels. The organic fillers, such as graphene oxide (GO), are used in the NaDC hydrogel systems. Wang *et al.* found that GO could effectively disperse in NaDC/PBS hydrogels, which enhanced the mechanical strength of the gels due to GO that could provide more binding sites of hydrogen bonds.^30^ Clearly, the properties of hydrogels are expected to be optimized by introducing the corresponding polymers. However, the intrinsic function and the mechanism of action for polymers in gelation are less involved and the understanding of the role of polymers in gelation and the corresponding mechanism of action is neither comprehensive nor systematic. Hence, further studies are still needed.

l-Aspartic acid (Asp) as a common amino acid can be used as an ammoniac detoxicating agent, hepar function accelerator, and fatigue refresher in pharmaceutical agents; also, it is widely utilized for mineral supplementation in the form of its potassium, magnesium or calcium salts.^31,32^ In this study, we combined the biological surfactant NaDC and amino acid Asp together to construct a gel system and investigated the corresponding gelation behavior in detail. The linear polymer poly(2-(2-methoxyethoxy)ethyl methacrylate-*co*-oligo(ethylene glycol)methacrylate) (P(MEO_2_MA_90_-*co*-OEGMA_10_)) and star-shaped polymer poly(2-(dimethylamino)ethyl methacrylate-*b*-2-(2-methoxyethoxy)ethyl methacrylate) (CDPDPM) were introduced into the NaDC/Asp hydrogels for exploring the effects of polymers on the properties of NaDC/Asp hydrogels systematically and the mechanism of polymer in gelation processes was proposed. The properties of the hydrogels were characterized by a variety of techniques, including polarized optical microscopy (POM), cryogenic scanning electron microscopy (cryo-SEM), X-ray powder diffraction (XRD), Fourier transform infrared (FT-IR) spectroscopy, and rheological measurements. Furthermore, the adsorption performances of hydrogels with and without polymers to methylene blue (MB) were studied using a UV-vis spectrometer. Our results may provide useful information not only for the adjustment of hydrogel properties on purpose, but also for the application of hydrogel in practice.

## 2. Materials and methods

### 2.1 Materials

**Fig. 1:**
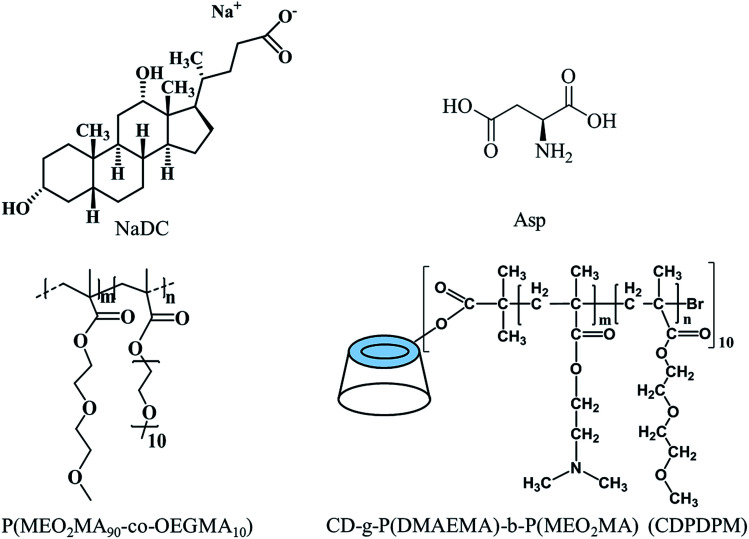
Chemical structures of NaDC, Asp, P(MEO_2_MA_90_-*co*-OEGMA_10_) and CDPDPM.

Sodium deoxycholate (NaDC) and l-aspartic acid (Asp) were purchased from J&K Scientific with 98% purity and used without further purification. Poly(2-(2-methoxyethoxy)ethyl methacrylate-*co*-oligo(ethylene glycol)methacrylate) (P(MEO_2_MA_90_-*co*-OEGMA_10_), *M*_n_ = 2.20 × 10^4^ g mol^−1^, PDI = 1.63) and poly(2-(dimethylamino)ethyl methacrylate-*b*-2-(2-methoxyethoxy)ethyl methacrylate) (CD-*g*-P(DMAEMA)_114_-*b*-P(MEO_2_MA)_115_, CDPDPM, *M*_n_ = 4.23 × 10^4^ g mol^−1^, PDI = 1.37) were synthesized *via* atom transfer radical polymerization (ATRP) according to our previous study (Fig. 1).^33,34^ Ultrapure water (with a resistivity of 18.2 MΩ cm) was used throughout the experiments.

### 2.2 Sample preparation

The 1 g L^−1^ solutions of the polymers were prepared first. Later, the stock solutions of NaDC and Asp were prepared by dissolving appropriate amounts of them in ultrapure water or polymer solutions. The solutions were mildly stirred at room temperature until all solids were dissolved. A series of sample solutions with desired concentrations of each component were prepared by mixing different amounts of stock solutions to a final volume of 5 mL. All samples were kept in a thermostatic incubator at 25.0 ± 0.1 °C for at least 4 weeks until the equilibrium state was attained and then, the phase behavior was inspected.

### 2.3 Identification and observation of birefringence

Photographs of sample birefringence were recorded using a Nikon Eclipse 50i pol polarizing optical microscope (POM) equipped with the Instec HCS302 heating/cooling stage.

### 2.4 Cryogenic scanning electron microscopy (cryo-SEM)

The microstructures of hydrosols and hydrogels were observed using cryo-SEM. The sample (5 μL) was loaded on the cryo-specimen holder and then transferred into a liquid nitrogen bath until liquid nitrogen ceased boiling. Then, the liquid nitrogen bath was transferred into the vacuum space for vacuuming immediately. After the liquid nitrogen ceased boiling, the specimen holder was transferred into the sample preparation cryo-cavity for sublimation (from −180 °C to −90 °C) for 4 min and then cooled to −120 °C for coating. The exposed surface topography was coated with a conducting deposit of gold within 1 min. The coated sample was then transferred at −120 °C into the lens of a field-emission scanning electron microscope (Hitachi S-4800, Japan) equipped with a cryo-stage (Gatan Alto-2500, UK) and observed under an accelerating voltage of 5 kV at a working distance of 19 mm.

### 2.5 Rheological measurements

The rheological measurements were carried out on the TA ARES rheometer equipped with a coni-cylinder geometry (cup diameter: 30 mm, bob diameter: 27 mm, bob length: 49 mm, gap: 1 mm). In oscillatory measurements, a dynamic strain sweep experiment at a fixed angular frequency of 6.28 rad s^−1^ was carried out prior to the subsequent frequency sweep in order to ensure that the selected strain was in a linear viscoelastic region. The viscoelastic properties of the samples were determined by oscillatory measurements in the angular frequency range of 0.1–100 rad s^−1^. The samples were measured at 25.0 ± 0.1 °C with a cyclic water bath. The samples remained in the coni-cylinder geometry for 10 min before measurement.

### 2.6 X-ray powder diffraction (XRD) measurements

The XRD patterns of the freeze-dried samples were recorded on the D/MAX-2550 VB/PC rotating anode X-ray powder diffractometer (Rigaku Corporation, Japan) with Cu Kα radiation (*λ* = 0.15418 nm) and a graphite monochromator. The samples were examined at room temperature over 1–10° in the 2*θ* mode (1° min^−1^).

### 2.7 Infrared spectroscopy

Fourier transform infrared (FT-IR) spectra were recorded using the Thermo Fisher Nicolet 6700 FT-IR spectrometer. The samples were prepared by KBr disc or film technique and the scan range was 4000–400 cm^−1^.

### 2.8 Adsorption study of methylene blue

For the typical time-dependent dye removal measurement, 1 mL hydrogels (100 mM NaDC/30 mM Asp in ultrapure water or polymer solutions) were first prepared and then, 3 mL methylene blue solution (0.025 mM) was added undisturbedly. The residual dye concentration after adsorption with hydrogel was measured using a UV-vis spectrophotometer (UV-2450, Shimadzu, Japan) at 25.0 ± 0.1 °C. All measurements were performed in a 1 cm-path length quartz cuvette in the wavelength range of 400–800 nm.

## 3. Results and discussion

### 3.1 Gelation behavior and properties of NaDC/Asp hydrogels

In order to obtain the information about the NaDC/Asp hydrogels, some experiments were carried out to characterize the gelation behavior and properties for the studied system.

#### 3.1.1 Phase behavior of NaDC/Asp system

**Fig. 2:**
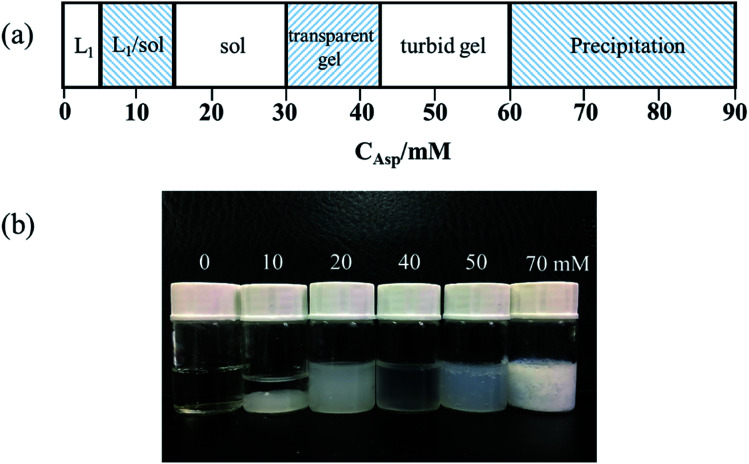
(a) Phase transition scheme with the addition of Asp to 100 mM NaDC solution at 25 ± 0.1 °C. (b) Photograph of typical samples. *C*_NaDC_ = 100 mM and *C*_Asp_ (from left to right) = 0 (L_1_ phase), 10 (L_1_/sol two phase), 20 (sol), 40 (transparent gel), 50 (turbid gel), and 70 (precipitation).

The phase behavior of the amino acid (Asp) with the facially amphiphilic bile salt (NaDC) in the aqueous solution was studied in detail. The phase boundaries were mainly delineated by visual observation and the minimum gelation concentration of Asp was confirmed by the inverted test tube method. The phase transition process of this system is examined at a fixed NaDC concentration of 100 mM, which is shown in Fig. 2(a). When the Asp concentration (*C*_Asp_) is below 5 mM, a transparent solution of micelles (L_1_ phase) is formed. With an increase in Asp (5 mM < *C*_Asp_ < 15 mM), the solution separates into biphasic L_1_/sol. On further increasing the Asp concentration, sol phase, transparent gel phase and turbid gel phase can be observed in succession. When the concentration of Asp is very high (above 60 mM), the gels are totally destroyed and precipitation is observed. The hydrogels are formed when the Asp concentration is between 30 mM and 60 mM. For the further application of the hydrogels, we focus on the transparent gel phase fabricated by 100 mM NaDC and 30 to 42 mM Asp.

#### 3.1.2 Microstructures of NaDC/Asp hydrogels

**Fig. 3:**
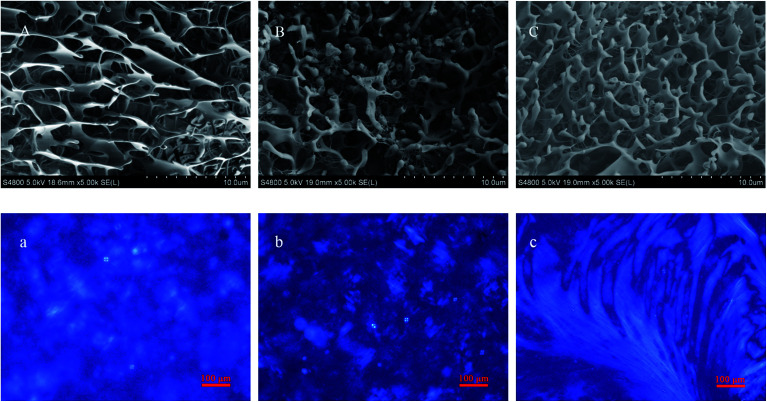
SEM (A–C) and POM (a–c) images of (A and a) 100 mM NaDC/20 mM Asp, (B and b) 100 mM NaDC/30 mM Asp, and (C and c) 100 mM NaDC/40 mM Asp.

For further study, the structure and gelation mechanism of hydrogels, the transparent gels and nearby sols were observed. Cryo-SEM and POM were used to obtain visual insights into the microscopic morphology of the NaDC/Asp hydrosols and hydrogels. SEM images (Fig. 3(A–C)) of the hydrosols and hydrogels display a compact structure composed of irregular networks. With an increase in Asp, the network becomes denser, suggesting that the mechanical strength of the hydrogels increases and correspondingly, the hydrosol transforms to a hydrogel. As shown in Fig. 3(a–c), a birefringence property of both NaDC/Asp hydrosols and hydrogels is observed through the POM observation. For all the studied samples, it can be noted that the samples show both Maltese crosses, which are characteristic of lamellar structures, and fan-like textures, which are characteristic of hexagonal structures. Moreover, with an increase in Asp, the birefringence property becomes stronger and Maltese crosses decrease, while fan-like textures increase, indicating the transition from lamellar structures to hexagonal structures. It can be concluded that the addition of Asp contributes to the formation of the hydrogels with better mechanical strength and stronger birefringence property.

#### 3.1.3 Rheological properties of NaDC/Asp hydrogels

**Fig. 4:**
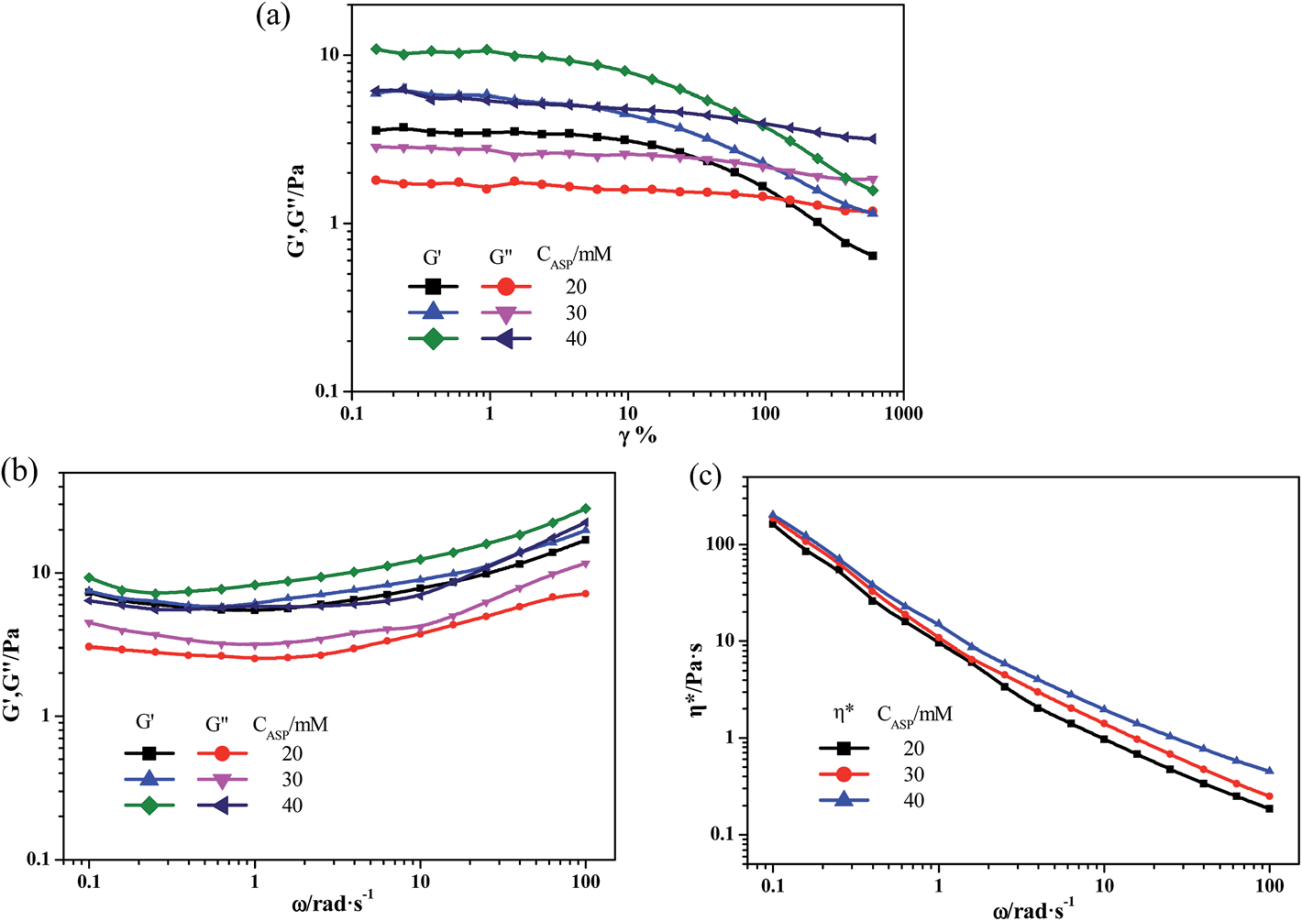
Rheological properties of NaDC/Asp hydrogels. (a) The elastic modulus *G*′ and the viscous modulus *G*′′ as a function of strain *γ* at 25 ± 0.1 °C under a fixed angular frequency *ω* = 6.28 rad s^−1^. Dynamic rheology ((b) *G*′ and *G*′′ and (c) complex viscosity η*) as a function of *ω* at 25 ± 0.1 °C under a constant *γ* of 5%. The contents of NaDC and Asp are: *C*_NaDC_ = 100 mM, and *C*_Asp_ = 20, 30, 40 mM, respectively.

Rheology is a key method to study the dynamic viscoelastic properties of hydrogel,^35,36^ which can provide detailed information about the mechanical properties of the hydrogels.^30^Fig. 4 summarizes the rheological properties of the hydrogels formed by 100 mM NaDC and Asp with different concentrations. Before the oscillatory frequency sweep, the applied strain should be checked to ensure that it is within the linear viscoelastic region, within which the elastic modulus (*G*′) is independent of the yield strain.^37^ If the applied strain is above a critical value (γ*), *G*′ will decrease rapidly due to the destruction of the microstructures of the hydrosols or hydrogels. As shown in Fig. 4(a), when NaDC concentration remains constant at 100 mM, the γ* value is approximately 10%, which is independent of the concentration of Asp. Moreover, the upward trend in *G*′ values indicates that increasing the concentration of Asp can make the network structures arrange more tightly. During the oscillatory rheological measurements (Fig. 4(b)), it is observed that both *G*′ and viscous modulus (*G*′′) increase with an increase in angular frequency for hydrosol formed in 100 mM NaDC/20 mM Asp. With the further increase in Asp concentration, hydrogels were formed and *G*′ was higher than *G*′′ in the entire studied angular frequency range and also, the hydrogels exhibit an elastic dominant property. Clearly, the typical “solid-like” rheological behavior is fully displayed.^38^ Furthermore, the results obtained by dynamic rheological measurements indicate that *G*′ and *G*′′ increase with an increase in Asp concentration along with a change in appearance from hydrosols to hydrogels, indicating the enhancement in the strength of network structures. The η* value of both hydrosols and hydrogels decreases with an increase in angular frequency, indicating shear-thinning behavior (Fig. 4(c)). Moreover, the value of η* increases with an increase in Asp concentration, which implies that the addition of Asp favors the enhancement of the mechanical property of NaDC/Asp hydrogels.

From the above experimental results, it can be inferred that for NaDC/Asp system, the addition of Asp can improve the mechanical properties of the hydrogels, which is in accordance with the results obtained from cryo-SEM. However, the ability of Asp to enhance the viscoelastic properties of the hydrogels is limited. The mechanical strength of network structures is still low, which limits the application of the NaDC/Asp hydrogels.

### 3.2 Effects of different polymers on the properties of NaDC/Asp hydrogels

Due to the poor mechanical properties of NaDC/Asp hydrogels, the polymer was introduced into the system. The additional polymer may participate in the network of hydrogels and the oxygen-containing functional groups of polymer may provide numerous binding sites for hydrogen bonding, which could benefit for the mechanical strength of the NaDC/Asp hydrogels. To improve the mechanical strength of the NaDC/Asp hydrogels, polymers with different structures including linear polymer P(MEO_2_MA_90_-*co*-OEGMA_10_) and star-shaped polymer CDPDPM (as shown in Fig. 1) were introduced into the NaDC/Asp system.

#### 3.2.1 Microstructures of NaDC/Asp/polymer hydrogels

**Fig. 5:**
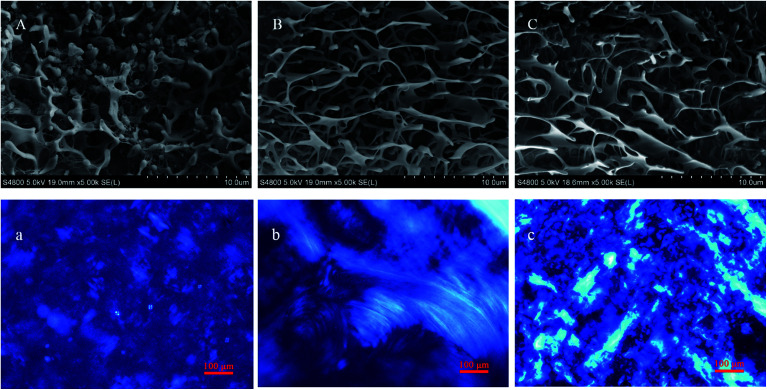
SEM (A–C) and POM (a–c) images of 100 mM NaDC/30 mM Asp (A and a) without polymer, (B and b) with 1 g L^−1^ P(MEO_2_MA_90_-*co*-OEGMA_10_), and (C and c) with 1 g L^−1^ CDPDPM.

The microstructures of NaDC/Asp/polymer hydrogels were observed through cryo-SEM and POM. The appearances of hydrogels formed in NaDC/Asp systems with and without polymers show no visible difference. From Fig. 5(A–C), cryo-SEM images show that all hydrogels display a compact structure composed of irregular networks, suggesting that the polymers can incorporate into the network structure without changing the structure of the hydrogels. Moreover, with the addition of polymer, the density of network structures increases remarkably, indicating that the mechanical strength of hydrogels increases. Correspondingly, POM images display that Maltese crosses disappear in the polymer-containing hydrogels. Instead, the polymer-containing hydrogels show the fan-like textures of hexagonal structures and the birefringence property of the hydrogels becomes stronger (Fig. 5(a–c)). It can be noted that the addition of polymer benefits the enhancement of both mechanical strength and birefringence property of the NaDC/Asp hydrogels. Furthermore, the star-shaped polymer CDPDPM performs better in optimizing the properties of the hydrogels than the linear polymer P(MEO_2_MA_90_-*co*-OEGMA_10_) because of the denser network structure and stronger birefringence property of the hydrogels.

#### 3.2.2 Rheological properties of NaDC/Asp/polymer hydrogels

**Fig. 6:**
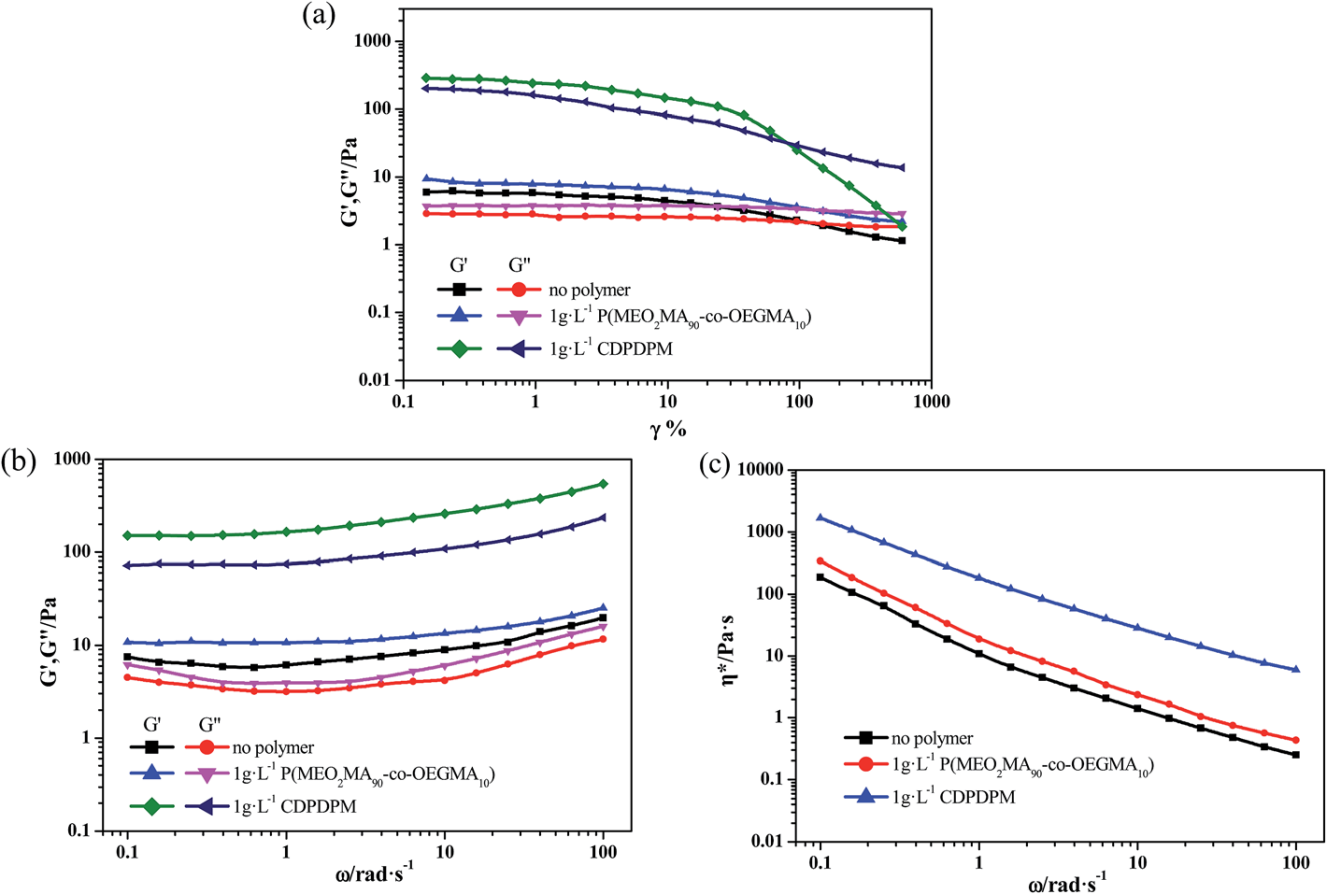
Rheological properties of NaDC/Asp hydrogels with and without polymers. (a) The elastic modulus *G*′ and the viscous modulus *G*′′ as a function of strain *γ* at 25 ± 0.1 °C under a fixed angular frequency *ω* = 6.28 rad s^−1^. Dynamic rheology ((b) *G*′ and *G*′′ and (c) complex viscosity η*) as a function of *ω* at 25 ± 0.1 °C under a constant *γ* of 5%. The contents of NaDC and Asp are: *C*_NaDC_ = 100 mM and *C*_Asp_ = 30 mM. The concentration of the polymer is 1 g L^−1^.

The mechanical properties of the hydrogels formed in NaDC/Asp/polymer systems were also investigated by dynamic rheological technique. Similar to the NaDC/Asp system, all the hydrogels predominantly exhibit elastic properties, for which *G*′ exceeds *G*′′ over the investigated angular frequency range shown in Fig. 6(b). Moreover, both hydrogels show a shear-thinning behavior; correspondingly, the η* decreases with an increase in angular frequency (Fig. 6(c)). However, the introduction of polymer improves the viscoelastic properties of hydrogels significantly. In stress sweep measurements (Fig. 6(a)), the γ* value is about 20% for polymer-containing hydrogels, which is larger than that observed in the NaDC/Asp system with γ* value of approximately 10%. Furthermore, the addition of polymer increases the *G*′ by different degrees. For the system containing linear polymer P(MEO_2_MA_90_-*co*-OEGMA_10_), the *G*′ is 10 Pa, which is slightly higher than that of NaDC/Asp hydrogel (5 Pa). However, for CDPDPM-containing hydrogel, the *G*′ value increases significantly and reaches 300 Pa, which is much higher than that of NaDC/Asp hydrogel. Furthermore, in oscillatory rheological measurements (Fig. 6(b)), both *G*′ and *G*′′ increase after the addition of the polymers. For P(MEO_2_MA_90_-*co*-OEGMA_10_)-containing hydrogel, *G*′ and *G*′′ are about 11 Pa and 5 Pa, respectively, which are slightly larger than the values for the hydrogel induced by NaDC and Asp with *G*′ and *G*′′ around 6 Pa and 4 Pa, respectively. However, after addition of the star-shaped polymer CDPDPM, *G*′ and *G*′′ significantly improve to 75 Pa and 154 Pa, respectively. Moreover, the value of η* increases drastically after the addition of polymer, particularly for the CDPDPM-containing hydrogel (Fig. 6(c)). These results indicate that the mechanical strength of the hydrogels can be improved by introducing a polymer. Moreover, the viscoelasticity of the CDPDPM-containing hydrogel is much higher than that of the P(MEO_2_MA_90_-*co*-OEGMA_10_)-containing hydrogel under the same condition, indicating that the star-shaped polymer CDPDPM performs better in strengthening the network structure of the hydrogels than the linear polymer P(MEO_2_MA_90_-*co*-OEGMA_10_).

#### 3.2.3 Adsorption performances to MB

Toxic dye-based industrial effluents are hazardous to both ecological balance and environment. Seeking convenient and efficient adsorbents for decontamination of waste waters is the goal that the researchers pursue. MB, an aromatic heterocyclic compound, is clinically used as the antidote of cyanide and nitrite poisoning as well as a photosensitizer to inactivate the virus in a single bag of fresh plasma. Therefore, MB is inevitably present in the medical sewage and the effective treatment of this type of medical sewage is significant to human health and environment.^39^ In this study, MB was selected as the object to explore the adsorption properties of NaDC/Asp/polymer hydrogels.

**Fig. 7:**
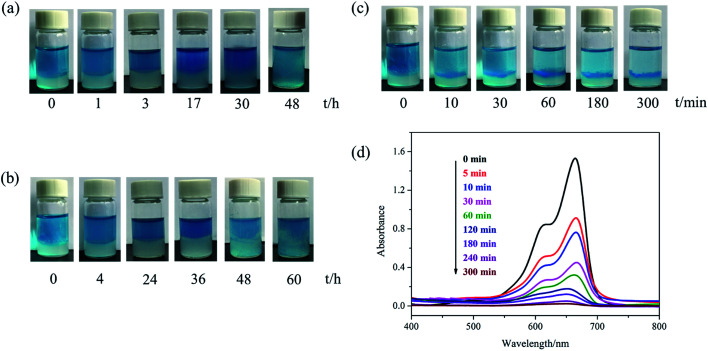
The pictorial diagrams of dye adsorption for 100 mM NaDC/30 mM Asp hydrogel (a) without polymer, (b) with 1 g L^−1^ P(MEO_2_MA_90_-*co*-OEGMA_10_), and (c) with 1 g L^−1^ CDPDPM. (d) UV-vis spectra of MB solution with time for the NaDC/Asp/CDPDPM hydrogel (*C*_NaDC_ = 100 mM, *C*_Asp_ = 30 mM and *C*_CDPDPM_ = 1 g L^−1^).

The gels fabricated by 100 mM NaDC/30 mM Asp mixed system with and without polymer were used for evaluating the dye removal behavior. Fig. 7 provides the pictorial diagrams of dye adsorption for different gel systems. It can be observed that the hydrogel formed in the NaDC/Asp system shows no adsorption ability to MB, but tends to capture water molecules of MB solution. Therefore, the hydrogel swells and dissolves within 48 h (Fig. 7(a)). Though the introduction of P(MEO_2_MA_90_-*co*-OEGMA_10_) increases the mechanical strength of the hydrogel slightly due to certain hydrophobicity, the hydrogel still swells after 4 h and collapses finally (Fig. 7(b)). However, for NaDC/Asp/CDPDPM hydrogel, the MB is efficiently entrapped inside the gel, gradually accompanied by the blue solution becomes clearer and clearer (Fig. 7(c)). Correspondingly, the variation in MB concentration in the aqueous solution was monitored by UV-vis spectra once it came in contact with the hydrogel (Fig. 7(d)). It is clearly shown that the absorbance decreases sharply after the MB aqueous solution comes in contact with the hydrogel and reaches stability after about 300 min. Moreover, it is worth noting that NaDC/Asp/CDPDPM hydrogel does not dissolve and almost does not swell after adsorption of MB, which makes the recycling of hydrogel possible. This may be due to the special structure that provides more binding sites for hydrogen bonding, resulting in a significant increase in the mechanical strength. Moreover, the stronger hydrophobic CDPDPM can incorporate into the hydrophobic microdomains to inhibit the entry of water, thus avoiding the swelling and dissolution of the hydrogels. It can be concluded that due to the efficient adsorption of the toxic dye molecules and recyclable property, the CDPDPM-containing hydrogel can be used as an environmentally friendly water-purifying agent.

### 3.3 Gelation mechanism

**Fig. 8:**
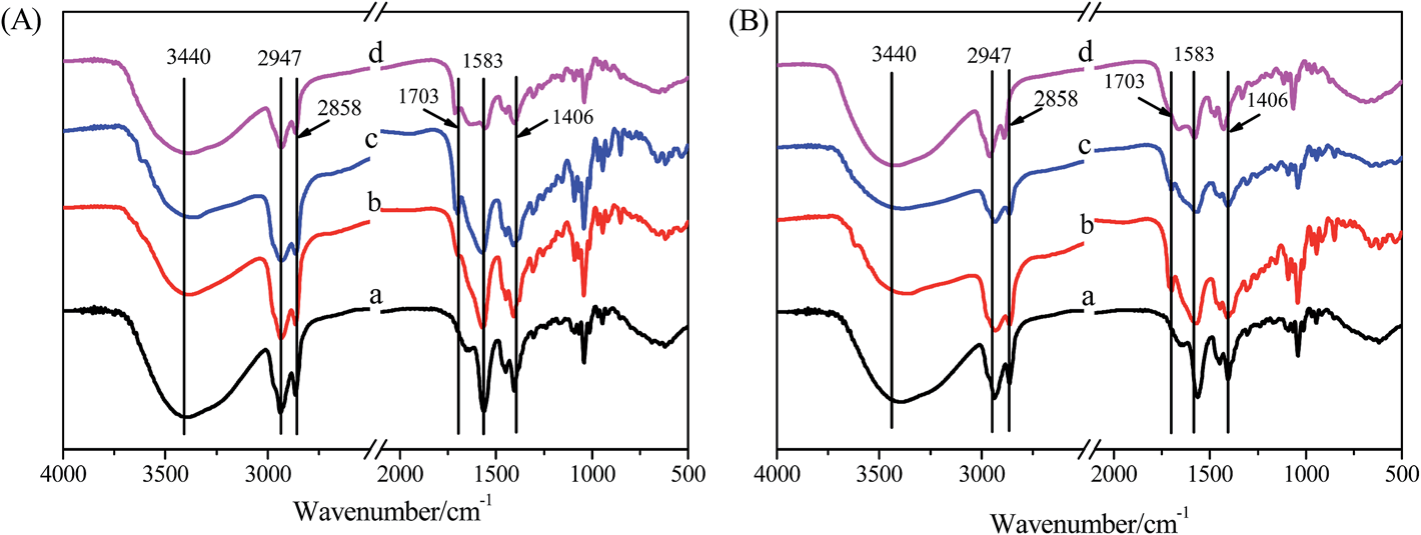
(A) FT-IR spectra of NaDC (a) and hydrogels of 100 mM NaDC with varied amounts of Asp: (b) 20, (c) 30, and (d) 40 mM. (B) FT-IR spectra of NaDC (a) and hydrogels of 100 mM NaDC/30 mM Asp (b) without polymer, (c) with 1 g L^−1^ P(MEO_2_MA_90_-*co*-OEGMA_10_), and (d) with 1 g L^−1^ CDPDPM.

For further studying the gelation mechanism of hydrogels formed in the NaDC/Asp/polymer system, FT-IR spectra were recorded as shown in Fig. 8. For all the samples, the wide peak at 3440 cm^−1^ is well known for being attributed to the symmetric and antisymmetric O–H stretching modes of NaDC. It can also be noted that the asymmetric and symmetric methylene stretching bands of NaDC are located at 2947 and 2858 cm^−1^, respectively. For pure NaDC (Fig. 8(A)) curve (a), there is no peak observed at around 1703 cm^−1^ (attributed to the symmetric stretching vibration of –COOH). For the hydrogels formed by 100 mM NaDC with varied amounts of Asp (Fig. 8(A)) curves (b–d), the peak appearing at 1583 cm^−1^ is assigned to the antisymmetric stretching vibration of –COO^−^, indicating the existence of the deprotonated form –COO^−^ in NaDC molecules.^40,41^ The bands at 1703, 1583, and 1406 cm^−1^ indicate that the carboxyl and carboxylate species coexist as aggregates in solutions,^21^ proving the existence of O–H⋯O hydrogen bonding between NaDC and deoxycholic acid or Asp molecules. For NaDC/Asp/polymer hydrogels (Fig. 8(B)) curves (c and d), all bands of the FT-IR spectra are similar to that of NaDC/Asp hydrogels under the same concentration (Fig. 8(B)) curve (b), indicating that the addition of polymer does not change the gelation route of NaDC/Asp hydrogels though it incorporates in the network structure of the hydrogels.

**Fig. 9:**
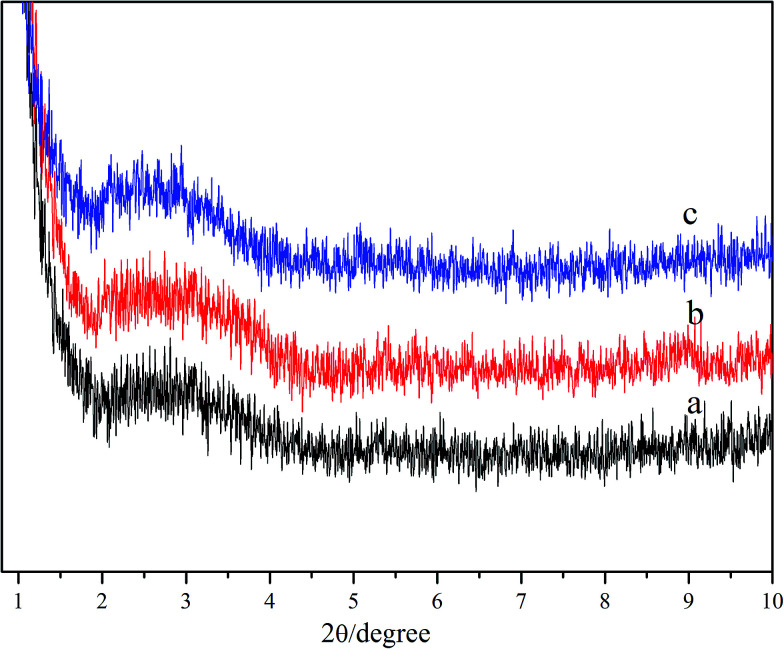
XRD patterns of 100 mM NaDC/30 mM Asp xerogels (a) without polymer, (b) with 1 g L^−1^ P(MEO_2_MA_90_-*co*-OEGMA_10_), and (c) with 1 g L^−1^ CDPDPM.

In order to get more detailed information about the studied hydrogels, XRD spectra were recorded to detect the mechanism of formation of the hydrogels.^23,26^ According to Bragg's law, the spacing distance (d) can be calculated from the reflection peaks, which are considered to be related to some cycled units induced by the arrangements of DC^−^ species. Fig. 9 shows the XRD spectra of hydrogels formed in the NaDC/Asp system with and without polymers. For hydrogel formed in 100 mM NaDC/30 mM Asp system, a strong peak centered at 2*θ* = 2.7 appears, from which the calculated *d* is 3.27 nm, which is slightly larger than twice that of the deoxycholate backbone length (1.5 nm × 2).^19^ Rich *et al.* have similarly reported that the diameter of deoxycholate complexes increases once other molecules such as amino acids or peptides appear in the solution during the formation of the complexes.^20^ The arrangement patterns of molecules in NaDC/Asp/polymer hydrogels were also investigated by XRD. As shown in Fig. 9, as compared to the hydrogel formed in the system without polymers, the *d* values of polymer-containing hydrogels show no visible change (3.29 nm for P(MEO_2_MA_90_-*co*-OEGMA_10_)-containing hydrogel and 3.32 nm for CDPDPM-containing hydrogel), indicating that the additional polymer either linear or star-shaped cannot change the style of the cycled units induced by the arrangement of DC^−^ species.

**Scheme 1:**
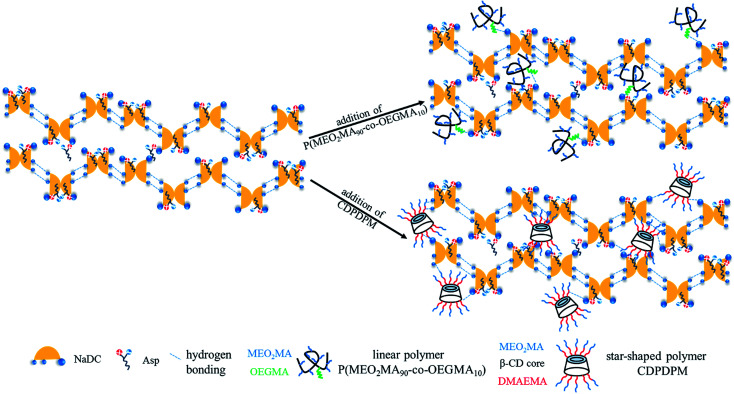
Schematic illustration of the gelation mechanism for NaDC/Asp/polymer hydrogel.

Based on the above results, the possible gelation mechanism of the NaDC/Asp/polymer hydrogels is proposed (Scheme 1). For NaDC/Asp hydrogel, the deoxycholate anions and amino acids may adopt lamellar arrangement, for which the hydrogen-bonded deoxycholate pairs are stacked together at their hydrophilic edges, allowing an opening angle between the planes of the two ring systems^42^ as shown in Scheme 1. In addition, two connected deoxycholate species form the continuous hydrophilic cavities and such interior cores act as the pocket, in which water molecules are held through interfacial tension.^41^ In fact, the process of the formation of hydrogels is complicated. The formation of this structure is driven by not only hydrogen bonding, but also hydrophobic interactions, electrostatic interactions, steric effects, and van der Waals forces. After the addition, the polymers can incorporate into the network of the hydrogels and correspondingly hydrogen bonds between the oxygen-containing functional groups of the polymer and the COO^−^ or OH of NaDC molecules will be formed. In this case, the polymer provides numerous binding sites, so that NaDC can easily connect with the polymer, leading to a drastic enhancement in the mechanical strength of the gels. Clearly, the special structure enables the CDPDPM to provide more binding sites for hydrogen bonding and the stronger hydrophobicity endows CDPDPM the ability to inhibit the entry of water, thus avoiding the swelling and dissolution of hydrogels. Therefore, the star-shaped polymer CDPDPM has more advantages in strengthening the network structure of the hydrogels than the linear polymer.

## 4. Conclusion

The gelation behavior and properties of NaDC with Asp in aqueous solution were investigated in detail. In addition, the linear polymer P(MEO_2_MA_90_-*co*-OEGMA_10_) and the star-shaped polymer CDPDPM were introduced into the NaDC/Asp hydrogels for exploring the effects of polymers on the properties of NaDC/Asp hydrogels systematically. The mechanism of gelation processes by polymers was also proposed. The results have indicated that the polymer can participate in the formation of a gel-network structure and provide numerous binding sites, so that NaDC can easily connect with the polymer, leading to an enhancement in the mechanical strength of the gels. Due to the special structure that provides more binding sites for hydrogen bonding and stronger hydrophobicity that inhibits the swelling and dissolution of hydrogels, the star-shaped polymer performs better in strengthening the network structure of the hydrogels than the linear polymer. Moreover, the introduction of the star-shaped polymer CDPDPM imparts additional functions to the composite hydrogels. The NaDC/Asp/CDPDPM hydrogel can adsorb MB efficiently without losing the gel state, thus making the recycling of hydrogel possible, while the NaDC/Asp hydrogel dissolves totally and NaDC/Asp/P(MEO_2_MA_90_-*co*-OEGMA_10_) hydrogel collapses in the MB aqueous solution. This indicates that the NaDC/Asp/CDPDPM hydrogel can be used as a novel and environmentally friendly material to recover MB from medical sewage. Moreover, the NaDC/Asp/CDPDPM hydrogel has potential to be used in drug delivery systems and bioengineering.

## Conflicts of interest

The authors declare no competing financial interest.

## Supplementary Material

RA-008-C8RA00171E-s001
