# Correction: Effects of polymers on the properties of hydrogels constructed using sodium deoxycholate and amino acid

**DOI:** 10.1039/c8ra90020e

**Published:** 2018-03-16

**Authors:** Yi Guo, Ruijin Wang, Yazhuo Shang, Honglai Liu

**Affiliations:** Key Laboratory for Advanced Materials, School of Chemistry & Molecular Engineering, East China University of Science and Technology Shanghai 200237 China shangyazhuo@ecust.edu.cn +86-21-64252767

## Abstract

Correction for ‘Effects of polymers on the properties of hydrogels constructed using sodium deoxycholate and amino acid’ by Yi Guo *et al.*, *RSC Adv.*, 2018, **8**, 8699–8708.

References 11 and 24 as published were incorrect; the correct versions are shown below as ref. 1 and 2, respectively.

The Royal Society of Chemistry apologises for these errors and any consequent inconvenience to authors and readers.

## Supplementary Material
